# Performance anxiety, wellbeing, and cultural factors in Chinese music conservatories: a two-study research of student experiences

**DOI:** 10.3389/fpsyg.2025.1671941

**Published:** 2025-10-27

**Authors:** Huiqin Bao, Lanying Yu

**Affiliations:** ^1^School of New Media and Arts, Dongguan City University, Dongguan, Guangdong, China; ^2^Conservatory of Music, Hunan University of Technology, Zhuzhou, Hunan, China

**Keywords:** performance anxiety, wellbeing, perfectionism, collectivism, music conservatory

## Abstract

**Introduction:**

Performance anxiety poses significant challenges for music conservatory students, especially in high-pressure educational contexts such as China. This research examined the link between performance anxiety and wellbeing, focusing on the moderating roles of perfectionism and collectivist orientation.

**Methods:**

Study 1, using a correlational design with 1,364 students, assessed these relationships within the PERMA model. Study 2 employed an experimental design with 218 students randomly assigned to individual success, collaborative success, or neutral conditions.

**Results:**

Results from Study 1 revealed that performance anxiety was negatively associated with wellbeing, with both perfectionism and collectivist orientation moderating this relationship. Study 2 showed that individual success conditions elicited the highest levels of anxiety, while collaborative settings offered partial support. Wellbeing outcomes were more complex: students in the individual condition reported higher wellbeing than those in the control group, whereas the collaborative condition did not differ significantly.

**Discussion:**

These findings suggest that perfectionism can intensify anxiety in individual contexts, while collectivist orientation and collaborative performance settings may provide some protective effects depending on context. The study offers critical insights for music education and highlights the need to balance performance pressure, social support, and cultural factors when designing interventions to promote wellbeing among conservatory students.

## Introduction

In China, music conservatories represent the pinnacle of classical music education, preparing students for competitive careers in performance, composition, and pedagogy. Previous research has shown that the intense dedication and rigorous training required can result in significant psychological stress, particularly performance anxiety (Hu, 2024). In a cultural context where academic and artistic excellence are closely tied to personal and family honor, failure in high-stakes performance settings has been reported to profoundly affect students’ mental wellbeing (Tai et al., 2018).

Understanding the psychological wellbeing of Chinese music students is crucial, given their unique educational environment shaped by Confucian values emphasizing collectivism, filial piety, and perseverance (Yang et al., 2022). While these values encourage prioritizing group success, conservatory training often emphasizes solo performances and individual excellence, creating tension between collectivist expectations and individualistic demands. This dynamic may influence how students experience and manage performance anxiety (Nwokenna et al., 2022). Two psychological factors are particularly relevant in this context: perfectionism and collectivist orientation. Perfectionism, common among high-achieving musicians, can motivate students but may also intensify anxiety through fear of failure and self-criticism (Kang and Li, 2018). In contrast, collectivist orientation, understood here as a cultural value rather than a performance format, has been suggested to provide social support and shared responsibility, thereby mitigating some of the isolating effects of performance pressure (Kirsner et al., 2023).

This study investigates the relationship between performance anxiety and wellbeing among Chinese conservatory students, focusing on the moderating roles of perfectionism and collectivist orientation. It employs two approaches: Study 1, a correlational investigation with a large student sample, and Study 2, an experimental study manipulating performance conditions to test causal relationships.

This research contributes significantly by providing a psychological perspective on Chinese music students, who have been underrepresented in global studies. Combining correlational and experimental methods identifies patterns in real-world experiences and tests causal mechanisms under controlled conditions. Its findings have important educational and cultural implications, guiding conservatories in developing culturally sensitive interventions that support mental health. By integrating performance and cultural psychology, this study enhances understanding of how Chinese music students navigate high-pressure environments, informing psychological research and educational practices to promote a balanced approach to music training in China.

### Theoretical support for this research

This research is grounded in established theories on performance anxiety, wellbeing, perfectionism, and cultural orientations. The following theoretical perspectives provide the foundation for understanding the relationships examined in this study.

Performance anxiety is a well-documented psychological phenomenon characterized by heightened stress and apprehension in high-stakes performance situations. Previous research has established that social pressure and perfectionism contribute significantly to increased anxiety levels (Kenny, 2011; Papageorgi et al., 2007). High expectations, whether from oneself or external evaluators, can lead to excessive self-monitoring, fear of failure, and physiological symptoms of anxiety, particularly in artistic and academic performance domains (Osborne and Kenny, 2005). Since music conservatories emphasize technical mastery and public performance, students are particularly vulnerable to performance anxiety due to constant evaluation and the pressure to meet exceptionally high standards.

Seligman’s (2011) PERMA model provides a comprehensive framework for understanding wellbeing by incorporating five key dimensions: Positive Emotion, Engagement, Relationships, Meaning, and Accomplishment. The model suggests that wellbeing is not merely the absence of distress but involves multiple facets contributing to an individual’s overall psychological health. Studies have shown that high levels of anxiety can negatively impact wellbeing by reducing positive affect, engagement in tasks, and feelings of accomplishment (Fredrickson and Joiner, 2002). In the context of this research, the PERMA model is applied to assess how performance anxiety influences different dimensions of wellbeing among music students.

Perfectionism is characterized by striving for flawlessness, setting excessively high standards, and engaging in critical self-evaluations (Flett and Hewitt, 2002). While adaptive perfectionism can be associated with motivation and high achievement, maladaptive perfectionism is linked to heightened anxiety, self-doubt, and reduced wellbeing (Stoeber and Otto, 2006). The relationship between perfectionism and anxiety is particularly relevant in performance-based disciplines such as music, where perfectionist tendencies may be reinforced by external evaluators and institutional culture. Previous studies have examined perfectionism as a potential moderator that may buffer or amplify the negative effects of performance anxiety on wellbeing (Huang et al., 2025; Mofield and Parker Peters, 2019).

Cultural orientation shapes individuals’ responses to stress, anxiety, and wellbeing. Individualism, which emphasizes personal achievement and self-reliance, is often associated with higher performance pressure and self-imposed perfectionism (Hogg et al., 2017). In contrast, collectivism, which prioritizes group harmony, social support, and shared responsibility, may protect against performance-related stress (Brown et al., 1992). Studies have found that individuals with strong collectivist orientations tend to experience lower anxiety levels and greater emotional support from their social networks, which can mitigate the impact of stressors (Markus and Kitayama, 1991; Oyserman et al., 2002). Given that this study examines music students in a Chinese conservatory setting—where collectivist cultural values are prevalent—it is essential to explore how collectivist orientation interacts with perfectionism and anxiety in predicting wellbeing.

This study integrates these theoretical perspectives to explore the relationships between performance anxiety, wellbeing, perfectionism, and cultural orientation among music students. By applying the PERMA model to measure wellbeing, considering perfectionism as a key individual difference factor, and incorporating cultural orientations, this research aims to provide a nuanced understanding of how these variables interact in a high-performance academic setting.

#### The current research

The primary objective of this research is to examine the relationships between performance anxiety, wellbeing, perfectionism, and collectivist orientation among music conservatory students. Specifically, the study aims to investigate how performance anxiety affects wellbeing as measured by the PERMA model and how perfectionism and collectivist orientation may moderate this relationship. By conducting both a correlational study (Study 1) and an experimental study (Study 2), this research seeks to provide a comprehensive understanding of the psychological factors influencing student experiences in high-pressure musical environments.

## Study 1

Study 1 examines both direct and moderated relationships between performance anxiety and wellbeing among music conservatory students. A *direct hypothesis* refers to the expected main effect of performance anxiety on wellbeing. In contrast, *moderation hypotheses* propose that this relationship may vary depending on the level of another variable, such as perfectionism or collectivist orientation. Drawing on the reviewed literature, the following hypotheses were formulated:

1. Direct Hypothesis: Performance anxiety is expected to be negatively associated with wellbeing, as assessed by the PERMA model, consistent with prior findings that anxiety diminishes positive affect and accomplishment (Fredrickson and Joiner, 2002; Seligman, 2011).
2. Moderation Hypothesis of Perfectionism: Perfectionism was expected to moderate the relationship between performance anxiety and wellbeing, such that higher levels of perfectionism could alter the strength or direction of this association, in line with research showing that perfectionism can function adaptively or maladaptively depending on context (Flett and Hewitt, 2002; Stoeber and Otto, 2006).
3. Moderation Hypothesis of Collectivist Orientation: Collectivist orientation was expected to moderate the relationship between performance anxiety and wellbeing, such that students with a strong collectivist orientation would experience a weaker negative effect of anxiety on wellbeing, as suggested by cultural psychology research emphasizing the buffering role of collectivist values (Markus and Kitayama, 1991; Oyserman et al., 2002).
4. Combined Moderation Hypothesis: The joint influence of perfectionism and collectivist orientation was expected to moderate the relationship between performance anxiety and wellbeing, with the possibility that their combined effect would shape how strongly anxiety impacts student wellbeing, building on evidence that multiple individual and cultural factors interact in shaping stress outcomes (Brown et al., 1992).

### Method

#### Participants

Study 1 included 1,364 music students from Guangdong and Hunan provinces, representing a range of ages and gender identities within the conservatory student population. Most participants (67.4%) were between 18 and 21 years old, which aligns with the typical undergraduate music conservatory enrollment in China. The most common age groups were 19 (19.3%) and 21 (25.6%), with fewer students represented in the older age groups. A smaller portion of the sample comprised graduate-level students, with 6.0% aged 26, 3.6% aged 27, and 3.7% aged 28. The presence of students up to 30 years old (2.6%) suggests the inclusion of individuals pursuing advanced degrees or extended training in professional performance programs.

The gender composition of the sample aligns with expected trends in Chinese conservatories, where female students tend to outnumber male students. In our sample, women comprised 70.5% (*n* = 961) and men 29.5% (n = 403). We report this descriptively and note that a female majority is broadly consistent with scholarship documenting the increasing participation of women in higher music education; however, conservatory-specific national benchmarks for a direct comparison are not available (Ho, 2023).

#### Procedure

The study received ethical approval in May 2024 from the Dongguan City University Research Ethics Committee (24-MS-11324), in line with institutional research ethics guidelines. Data were collected in Guangdong and Hunan provinces from music conservatories, university music departments, and vocational arts schools. This allowed us to include a wide range of students engaged in performance-focused training. Recruitment took place through printed notices posted on institutional boards and digital invitations distributed via WeChat groups, QQ student forums, and official online pages of the participating institutions.

Participants completed the survey online using a smartphone-compatible platform, which made participation flexible and convenient. Before beginning, each student read and signed an informed consent form that described the aims of the study, the voluntary nature of participation, and the confidentiality measures in place. Participants also consented to the use of their anonymized data in scientific publications. They were informed that they could withdraw at any time without penalty. Responses to sensitive measures, such as anxiety and wellbeing, were collected anonymously. Data were stored on secure, password-protected servers accessible only to the research team. No personal identifiers (e.g., names or student ID numbers) were collected, and analyses were conducted on aggregated data.

To encourage participation, reminder messages were sent through institutional bulletin boards, general mailing lists, and official student social media groups (e.g., WeChat and QQ forums). These reminders were never sent directly to individual students, which ensured that no personal contact information was recorded or used. Data collection took place between October and November 2024. In describing the educational context, we specifically refer to Chinese music conservatories and university-level programs in music. The multi-institutional and multi-platform approach helped us reach a broad cross-section of music students while maintaining confidentiality and ethical integrity.

#### Instruments

Performance anxiety was measured using the Performance Anxiety Inventory (Kenny, 2011), a widely used instrument designed to assess individuals’ emotional, cognitive, and physiological responses to performance situations. Participants rated their agreement with statements regarding their experiences of anxiety in high-stakes performance settings using a 5-point Likert scale (1 = Strongly Disagree to 5 = Strongly Agree). Higher scores indicated greater levels of performance anxiety. The internal consistency of this measure was Cronbach’s alpha of 0.83.

*Wellbeing* was assessed using the PERMA Profiler (Seligman, 2011), which measures the five core dimensions of Positive Emotion, Engagement, Relationships, Meaning, and Accomplishment. Each domain was evaluated through three items, and an overall PERMA wellbeing score was computed as the average of all responses. Participants rated their agreement on a 5-point Likert scale (1 = Never to 5 = Always). This measure has been widely used in positive psychology research and has demonstrated strong reliability in the present study (Cronbach’s alpha 0.80).

*Perfectionism* was measured using the Frost Multidimensional Perfectionism Scale (Frost et al., 1990). This 35-item questionnaire assesses six dimensions of perfectionism: Concern over Mistakes, Doubts about Actions, Personal Standards, Parental Expectations, and Parental Criticism. Organization. Responses were recorded on a 5-point Likert scale (1 = Strongly Disagree to 5 = Strongly Agree), with higher scores reflecting greater perfectionistic tendencies. The Cronbach’s *α* for the full scale was 0.92, indicating high internal consistency for assessing perfectionistic traits in music students.

*Collectivist orientation* was evaluated using eight items adapted from the Individualism–Collectivism Scale (Singelis et al., 1995). Items focused on perceptions of group harmony, loyalty, and shared responsibility in musical performance settings. Sample statements included:

“The values emphasized in my learning environment align with the importance of group harmony and loyalty.” Participants rated their agreement on a 7-point Likert scale (1 = Strongly Disagree to 7 = Strongly Agree). In this study, Cronbach’s α for the scale was 0.89, indicating high reliability and consistency in measuring collectivist value orientation.

Participants also provided demographic details, including age, gender, and academic year.

### Results

#### Descriptive and correlational analyses

Means (*M*) and standard deviations (*SD*) were calculated for all variables. As shown in Table 1, the mean score for performance anxiety was 2.64 (*SD* = 0.87), while the mean score for wellbeing was 3.00 (*SD* = 1.47). Perfectionism had a mean score of 3.79 (*SD* = 0.80), and collectivist orientation had a mean score of 3.62 (*SD* = 0.86).

**Table 1:** Descriptive statistics and Pearson’s correlations for key variables.

Variable	*M*	*SD*	Performance anxiety	Wellbeing	Perfectionism	Collectivist orientation
1. Performance anxiety	2.64	0.87	–			
2. Wellbeing	3.00	1.47	−0.084**	–		
3. Perfectionism	3.79	0.80	−0.151**	0.387**	–	
4. Collectivist orientation	3.62	0.86	0.148**	−0.134**	−0.233**	–

*N* = 1,364. **p < 0.01.

Pearson’s correlation analysis revealed significant relationships among the variables. Performance anxiety was negatively correlated with wellbeing (*r* = −0.084, *p* = 0.002), suggesting that higher anxiety is associated with lower wellbeing. Performance anxiety was also negatively correlated with perfectionism (*r* = −0.151, *p* < 0.001), but positively correlated with collectivist orientation (*r* = 0.148, *p* < 0.001). Wellbeing showed a significant positive correlation with perfectionism (*r* = 0.387, *p* < 0.001) and a significant negative correlation with collectivist orientation (*r* = −0.134, *p* < 0.001). Finally, perfectionism and collectivist orientation were negatively correlated with one another (*r* = −0.233, *p* < 0.001).

### Hypotheses testing

The results of Study 1, which employed Igartua and Hayes (2021) PROCESS Model 2, are presented below, organized according to the hypotheses tested. This study investigated the direct relationship between performance anxiety and wellbeing, as well as the moderating roles of perfectionism and collectivist orientation, individually and in combination.

#### Direct hypothesis: relationship between performance anxiety and wellbeing

It was hypothesized that there would be a negative relationship between performance anxiety and wellbeing, as assessed by the PERMA model. A linear regression analysis supported this hypothesis, showing that performance anxiety significantly predicted wellbeing (*b* = −1.18, SE = 0.30, *t* = −3.99, *p* < 0.001). This regression analysis was conducted after the correlational analysis to test predictive relationships more directly. These findings indicate that higher levels of performance anxiety are associated with lower levels of wellbeing.

#### Moderation hypothesis of perfectionism

The second hypothesis proposed that perfectionism would moderate the relationship between performance anxiety and wellbeing. The interaction term (performance anxiety × perfectionism) was significant (*b* = 0.42, SE = 0.05, *t* = 8.26, p < 0.001), indicating a moderating effect. A simple slopes analysis showed that at low levels of perfectionism, performance anxiety had a strong negative effect on wellbeing (*b* = −0.54, *p* < 0.001). At high levels of perfectionism, the relationship was positive and significant (*b* = 0.42, *p* < 0.001). This simple slopes analysis clarifies how the effect of performance anxiety on wellbeing changes depending on the level of perfectionism, rather than implying a protective “buffer.” These findings indicate that perfectionism influenced the direction of the relationship, reducing or even reversing the negative association between performance anxiety and wellbeing in this sample.

#### Moderation hypothesis of collectivist orientation

The third hypothesis predicted that the negative relationship between performance anxiety and wellbeing would be less pronounced among individuals with a strong collectivist orientation. This hypothesis was also supported, as the interaction term (performance anxiety × collectivist orientation) was significant (*b* = −0.13, SE = 0.05, *t* = −2.80, *p* = 0.005). The results indicate that individuals with a strong collectivist orientation experience a weaker negative association between performance anxiety and wellbeing compared to those with a lower collectivist orientation.

#### Combined moderation hypothesis

The final hypothesis proposed that perfectionism and collectivist orientation would jointly moderate the relationship between performance anxiety and wellbeing. The three-way interaction was significant, explaining additional variance in wellbeing (R-squared change = 0.0557, *F*(2, 1,358) = 47.79, *p* < 0.001). A conditional effects analysis was conducted to examine how the strength of the relationship varied depending on the joint levels of perfectionism and collectivist orientation. When both perfectionism and collectivist orientation were low, performance anxiety strongly predicted lower wellbeing (*b* = −0.54, *p* < 0.001). By contrast, when both were high, the relationship between performance anxiety and wellbeing was not significant (*b* = 0.13, *p* = 0.113). These results suggest that the combined presence of high perfectionism and strong collectivist orientation changed the strength of the association, weakening the negative effect of performance anxiety on wellbeing.

### Discussion of Study 1 findings

This research offers important insights into the psychological experiences of music conservatory students, particularly regarding the impact of performance anxiety on wellbeing. Consistent with prior studies (Kenny, 2011; Papageorgi et al., 2007), Study 1 identified a significant negative association between performance anxiety and wellbeing, supporting the notion that higher anxiety levels are linked to reduced wellbeing. This finding aligns with the PERMA model, which emphasizes how stress and anxiety can diminish positive emotions, engagement, and a sense of accomplishment (Rice, 2024).

The moderation analyses further highlighted the roles of perfectionism and collectivist orientation. In this sample, our findings showed that higher levels of perfectionism appeared to reduce or even reverse the negative impact of performance anxiety on wellbeing, suggesting that, under certain conditions, striving for high standards can serve as a source of motivation rather than distress. By contrast, previous research has shown that extreme or maladaptive forms of perfectionism may heighten anxiety when closely tied to self-worth (Flett and Hewitt, 2002). This dual perspective suggests that perfectionism is not uniformly a risk factor but may function differently depending on the balance between adaptive and maladaptive components. Collectivist orientation moderated the anxiety–wellbeing relationship in a different way. Students with stronger collectivist values experienced a weaker negative association, indicating that social belonging and shared responsibility may provide emotional support that mitigates the burden of individual performance anxiety (Markus and Kitayama, 1991). These findings underscore the importance of considering cultural context when examining performance-related stress.

## Study 2

Study 2 employs an experimental design to further investigate how performance pressure and perfectionism influence students’ psychological outcomes. The three experimental conditions (high-pressure individual success, high-pressure collaborative success, and a neutral condition) were developed for the present study, drawing conceptually on prior literature on performance pressure, collectivist versus individualist orientations, and perfectionism. The study compares performance anxiety levels among students in three conditions: high-pressure individual success, high-pressure collaborative success, and a neutral condition. It also assesses how these conditions affect wellbeing, hypothesizing that collaborative success conditions may provide greater psychological support than individual ones. Furthermore, Study 2 examines whether the interaction between performance anxiety and perfectionism differs across conditions, and whether perfectionism exerts a stronger influence in individual than in collective contexts. Finally, it tests whether the neutral condition is associated with lower anxiety and higher wellbeing compared to the high-pressure conditions. Based on the reviewed literature, the following hypotheses were formulated:

1. Hypothesis on Anxiety: Students in the high-pressure individual condition, emphasizing perfectionism, were expected to report higher levels of performance anxiety compared to those in the high-pressure collaborative condition with the same emphasis, consistent with findings that individualistic performance contexts exacerbate anxiety (Kenny, 2011; Osborne and Kenny, 2005).
2. Hypothesis on Wellbeing: Wellbeing, as measured by the PERMA model, was expected to be lower in the high-pressure individual condition emphasizing perfectionism than in the high-pressure collaborative condition, due to the perceived support in the collaborative setting. Although collaborative performance is not identical to collectivist cultural orientation, it conceptually parallels research suggesting that shared responsibility and group support—key features of collectivism—can mitigate performance stress (Markus and Kitayama, 1991; Oyserman et al., 2002).
3. Hypothesis on Perfectionism Interaction: The interaction between performance anxiety and perfectionism was hypothesized to differ across conditions, with high perfectionism expected to amplify anxiety and reduce wellbeing more strongly in the individual condition compared to the collaborative condition. This expectation draws on prior work linking perfectionism with heightened vulnerability in solo performances (Flett and Hewitt, 2002; Stoeber and Otto, 2006).
4. Neutral Condition Comparison: The neutral condition, which lacks emphasis on perfectionism, was expected to be associated with lower anxiety and higher wellbeing compared to both high-pressure conditions. This hypothesis reflects studies showing that absence of evaluative pressure is associated with lower anxiety and higher wellbeing (Fredrickson and Joiner, 2002).

### Method

#### Participants

Study 2 involved 218 music conservatory students who were assigned to one of three experimental conditions: a control group (*n* = 88), a collaborative success condition (*n* = 69), or an individual success condition (*n* = 61). The students were drawn from the same academic environment as Study 1, ensuring consistency in participant characteristics.

The age distribution of participants ranged from 18 to 22 years old, with a relatively even spread across age groups. The largest age group was 22 years old (22.9%), followed by 18 years old (21.1%), 21 years old (20.2%), 19 years old (18.3%), and 20 years old (17.4%). This distribution indicates that most participants were undergraduate students, reflecting the typical age range for conservatory training in China. Study 2 had mostly male participants (57.3%), with females comprising 42.7%. The gender distribution observed in Study 2 could be related to the composition of conservatory specializations, as some fields (e.g., instrumental training, conducting, composition) are often more male-dominated. However, because information on participants’ specific specializations was not collected, this explanation remains tentative and cannot be verified with the present data.

#### Procedure

The study followed the principles of the Declaration of Helsinki and received approval from the Dongguan City University Research Ethics Committee (24-MS-11324). Participants were randomly assigned to one of three experimental conditions that varied in performance pressure and perfectionism expectations: (a) high-perfectionism individual success, where students imagined being evaluated by a demanding jury expecting flawless execution; (b) high-perfectionism collaborative success, where they imagined an ensemble performance with shared responsibility for perfection; and (c) a neutral scenario, where they imagined a routine practice session without evaluation. For clarity, in this study the term collaborative success refers exclusively to the ensemble performance condition in the experiment, where responsibility was shared among group members. It was conceptually inspired by, but is not equivalent to, collectivism as a broader cultural orientation.

Recruitment was conducted through physical notices posted on campus and digital announcements shared on institutional channels. These included official university webpages, WeChat groups for music students, QQ discussion forums, and online communities focused on conservatory life. This approach ensured that students from a variety of performance backgrounds were reached.

Before participation, students reviewed and signed an informed consent form that emphasized voluntariness, confidentiality, and the right to withdraw at any point without consequence. Participants also consented to the use of their anonymized data in scientific publications. Responses to sensitive measures such as anxiety and wellbeing were collected anonymously through Qualtrics. No personal identifiers were requested, and all data were stored on secure, password-protected servers accessible only to the research team. Analyses were conducted at the group level, ensuring that individual students could not be identified.

Data collection took place in November 2024 during the academic semester, providing a realistic context for student experiences. The online format allowed for rigorous randomization, included a manipulation check to verify the effectiveness of the scenarios, and safeguarded participant welfare through anonymity and secure handling of the data.

#### Instruments

Performance anxiety and Wellbeing were measured using the same instruments as in Study 1. The internal consistency of the Performance Anxiety Inventory in this study was a Cronbach’s alpha of 0.85. The PERMA Profiler has demonstrated strong reliability in the present study (Cronbach’s alpha 0.89).

#### Manipulation check: perceived perfectionism and social pressure

To verify the effectiveness of the experimental manipulation, participants completed two separate scales designed to measure their perception of perfectionism and social pressure within their assigned condition. Both questions were developed for this study and the text of each item is included in the next section.

*Perceived Perfectionism*: Participants responded to the question, “To what extent did you feel that the situation required you to perform perfectly, with no mistakes allowed?” using a 5-point Likert scale (1 = Not at all, 5 = Extremely). This measure was included to confirm that the high-perfectionism conditions successfully elicited stronger perceptions of perfectionism than the neutral condition.

*Perceived Social Pressure*: To assess the extent to which participants felt external pressure, they responded to the question, “To what extent did you feel external pressure to meet high expectations in this situation?” using the same 5-point Likert scale. This measure was included to determine whether participants in the high-pressure conditions reported greater social pressure than those in the neutral condition.

#### Experimental condition stimuli

Participants were randomly assigned to one of three experimental conditions designed to manipulate the level of performance pressure and perfectionism emphasis:

*High Perfectionism, Individual Success Condition:* Participants imagined preparing for an individual performance in which their execution was closely scrutinized by a demanding jury expecting flawless precision. The scenario emphasized the high stakes of evaluation and the negative consequences of making mistakes, reinforcing an intense, self-focused pressure to achieve perfection.

*High Perfectionism, Collaborative success Condition:* Participants imagined participating in a group performance where the entire ensemble’s success depended on each member’s technical precision. This condition emphasized shared responsibility and collective pressure, making performance perfection a collaborative expectation rather than an individual burden.

*Neutral Condition:* Participants envisioned attending a routine practice session in which no explicit expectations for perfection were imposed. Unlike the high-pressure conditions, this scenario framed performance as an opportunity for improvement without judgment or evaluation.

These three conditions were developed specifically for the present study, drawing conceptual inspiration from prior research on performance pressure, perfectionism, and cultural orientations, but operationalized here in a novel way.

### Results

#### Manipulation check

The effectiveness of the experimental manipulation was examined with one-way analyses of variance (ANOVAs) on the Perceived Perfectionism and Perceived Social Pressure scales, comparing the control group, the collaborative success condition, and the individual success condition. Descriptive statistics are presented in Table 1 (Perceived Perfectionism) and Table 2 (Perceived Social Pressure), along with the ANOVA results and *post-hoc* comparisons.

**Table 2:** Conditional effects of performance anxiety on wellbeing at different levels of perfectionism and collectivist orientation.

Perfectionism (W)	Collectivist orientation (Z)	Effect of performance anxiety (b)	Standard error (SE)	*t*-value	*p*-value	95% confidence interval (lower, upper)
3.0	2.6	−0.25	0.08	−3.00	0.003	−0.42, −0.09
3.0	3.8	−0.41	0.06	−6.98	<0.001	−0.53, −0.30
3.0	4.8	−0.54	0.07	−7.53	<0.001	−0.68, −0.40
3.8	2.6	0.08	0.07	1.30	0.195	−0.04, 0.21
3.8	3.8	−0.07	0.04	−1.74	0.082	−0.16, 0.01
3.8	4.8	−0.20	0.07	−3.04	0.002	−0.34, −0.07
4.6	2.6	0.42	0.07	6.22	<0.001	0.29, 0.56
4.6	3.8	0.27	0.06	4.56	<0.001	0.15, 0.38
4.6	4.8	0.13	0.08	1.59	0.113	−0.03, 0.30

For Perceived Perfectionism, the omnibus ANOVA was significant, *F*(2, 215) = 3.33, *p* = 0.038, indicating that at least one group differed from the others. Tukey B *post-hoc* comparisons showed that the control group reported significantly lower perceptions of perfectionism than both the collaborative and the individual success groups (*p* < 0.05). No significant difference was found between the two high-perfectionism conditions (*p* > 0.05).

Together, these results confirm that the manipulation was effective: both high-perfectionism conditions produced higher perceptions of perfectionism than the control group, while the individual success condition elicited the highest perceptions of social pressure, followed by the collaborative condition and then the control group.

### Hypotheses testing

Univariate analysis of variance (ANOVA) was conducted to examine the effects of experimental conditions (control group, collaborative success, individual success) and perfectionism on performance anxiety. The model explained 57.6% of the variance in performance anxiety (*R*^2^ = 0.576), although the adjusted R^2^ was 0.214, reflecting the complexity and the relatively large number of levels for perfectionism in the design. Table 1 presents the descriptive statistics and the ANOVA results.

#### *Hypothesis 1*: performance anxiety across conditions

The first hypothesis proposed that performance anxiety would be higher in the individual condition compared to the collaborative condition. ANOVA results showed significant differences in anxiety across groups, *F*(2, 217) = 8.258, *p* < 0.001, partial η^2^ = 0.124. *Post-hoc* comparisons indicated that students in the individual success condition (*M* = 4.18, *SD* = 0.79) reported significantly higher anxiety than those in the control group (*M* = 3.74, *SD* = 0.69, p < 0.001). Anxiety scores in the individual condition were also marginally higher than in the collaborative condition (*M* = 3.93, *SD* = 0.57, *p* = 0.071). No significant difference was observed between the control and collaborative conditions (*p* = 0.131). These findings provide partial support for the hypothesis, with the clearest difference emerging between the individual and control groups. A summary of descriptive statistics and *post-hoc* analyses for performance anxiety is presented in Table 3.

**Table 3:** Means, standard deviations, and one-way ANOVA for perceived perfectionism by condition.

Condition	*n*	*M*	*SD*	95% CI [LL, UL]	Minimum	Maximum
Control group	88	42.71	12.19	[40.13, 45.29]	22.44	75.44
Collaborative success	69	47.34	12.51	[44.33, 50.34]	24.44	72.44
Individual success	61	47.49	15.17	[43.60, 51.37]	25.44	75.44
Total	218	45.51	13.33	[43.73, 47.29]	22.44	75.44

ANOVA source	SS	*df*	MS	*F*	*p*
Between groups	1158.58	2	579.29	3.33	0.038
Within groups	37379.01	215	173.86		
Total	38537.59	217			

*n*, sample size; M, mean; SD, standard deviation; CI, confidence interval; LL, lower limit; UL, upper limit.

A similar ANOVA was conducted for Perceived Social Pressure, yielding a large effect, *F*(2, 215) = 660.26, *p* < 0.001. *Post-hoc* tests indicated that all three groups differed significantly (*p* < 0.05). The control group reported the lowest levels of perceived social pressure, followed by the collaborative success condition, and the individual success condition reported the highest levels.

#### *Hypothesis 2*: wellbeing across conditions

The second hypothesis stated that wellbeing would be lower in the individual condition compared to the collaborative condition. ANOVA results showed significant differences across groups, *F*(2, 217) = 7.415, *p* < 0.001, partial *η*^2^ = 0.112. *Post-hoc* tests indicated that students in the individual success condition (*M* = 14.70, *SD* = 3.09) reported significantly higher wellbeing than those in the control group (*M* = 13.11, *SD* = 2.70, p < 0.001) (Tables 4, 5). Wellbeing scores in the collaborative condition (*M* = 13.90, *SD* = 2.52) did not differ significantly from either the control group (*p* = 0.061) or the individual condition (*p* = 0.085). These findings do not align fully with the hypothesis, but they suggest that the collaborative condition may have provided moderate support for wellbeing, while the individual condition was unexpectedly associated with higher scores than the control group. Table 6 provides the descriptive statistics and *post-hoc* comparisons for wellbeing.

**Table 4:** Means, standard deviations, and one-way ANOVA for perceived social pressure by condition.

Condition	*n*	*M*	*SD*	95% CI [LL, UL]	Minimum	Maximum
Control group	88	109.40	5.26	[108.29, 110.52]	101.55	112.88
Collaborative success	69	129.31	5.68	[127.94, 130.67]	124.21	135.55
Individual success	61	151.90	10.04	[149.33, 154.47]	146.88	180.88
Total	218	127.59	18.71	[125.09, 130.09]	101.55	180.88

ANOVA source	SS	*df*	MS	*F*	*p*
Between groups	65350.18	2	32675.09	660.26	< 0.001
Within groups	10640.02	215	49.49		
Total	75990.21	217			

*n*, sample size; M, mean; SD, standard deviation; CI, confidence interval; LL, lower limit; UL, upper limit.

**Table 5:** Descriptive statistics and *post-hoc* comparisons for performance anxiety across conditions.

Experimental condition	*M*	*SD*	*N*
Control group	3.74	0.69	88
Collaborative success	3.93	0.57	69
Individual Success	4.18	0.79	61

Condition pair	Mean difference	*p*-value	95% CI (lower, upper)
Control vs. individual	−0.44	< 0.001	−0.69, −0.19
Control vs. collaborative	−0.20	0.131	−0.44, 0.04
Collaborative vs. individual	−0.25	0.071	−0.51, 0.02

**Table 6:** Descriptive statistics and *post-hoc* comparisons for wellbeing across conditions.

Experimental condition	*M*	*SD*	*N*
Control group	13.11	2.70	88
Collaborative success	13.90	2.52	69
Individual success	14.70	3.09	61

Condition pair	Mean difference	*p*-value	95% CI (lower, upper)
Control vs. individual	−1.59	< 0.001	−2.43, −0.75
Control vs. collaborative	−0.78	0.061	−1.60, 0.03
Collaborative vs. individual	−0.80	0.085	−1.69, 0.09

#### *Hypothesis 3*: interaction between perfectionism and performance anxiety

The third hypothesis predicted that perfectionism would interact with condition to influence anxiety and wellbeing. Results supported this expectation. For anxiety, a significant interaction was found, *F*(98, 117) = 1.439, *p* = 0.030, partial *η*^2^ = 0.547. Students high in perfectionism reported the highest anxiety in the individual success condition compared to the collaborative and control groups. A significant interaction also emerged for wellbeing, *F*(98, 117) = 2.489, *p* < 0.001, partial *η*^2^ = 0.676. Students high in perfectionism reported lower wellbeing in the individual condition than in the collaborative condition. These results indicate that perfectionism intensified the negative effects of the individual condition.

#### *Hypothesis 4*: neutral condition comparison

The final hypothesis proposed that the neutral condition would produce lower anxiety and higher wellbeing compared to the high-pressure conditions. Results provided partial support. For anxiety, students in the control group reported significantly lower scores than those in the individual success condition (*p* < 0.001), but no difference compared to the collaborative condition (*p* = 0.131). For wellbeing, students in the control condition scored lower than those in the individual condition (*p* < 0.001), while differences between the control and collaborative conditions were not significant (*p* = 0.061). Overall, the neutral condition did not consistently show the predicted benefits across outcomes.

## Discussion of Study 2 findings

Study 2 experimentally examined how different performance conditions influence anxiety and wellbeing. Students in the high-pressure individual condition reported the highest levels of anxiety, particularly when performance was framed around perfectionistic standards, which is consistent with research showing that individual responsibility heightens stress in evaluative contexts (Osborne and Kirsner, 2022). Differences between the individual and collaborative conditions, however, were less pronounced than expected, with anxiety levels in the collaborative condition falling between those of the control and individual groups.

The interpretation of wellbeing outcomes requires particular nuance. Although it was hypothesized that students in the individual success condition would show lower wellbeing than those in the collaborative condition, the results indicated a different pattern. Students in the individual condition reported significantly higher wellbeing than those in the control group, while the collaborative condition did not differ significantly from either group. One possible explanation is that highly demanding individual performance contexts, despite elevating anxiety, may also provide students with a sense of achievement or validation that supports their wellbeing. By contrast, the collaborative condition appeared to offer moderate support but did not yield significant differences compared to the other groups. These findings suggest that performance contexts can influence wellbeing in more complex ways than expected, highlighting the need to consider both the stress and the potential motivational benefits of individual performance settings.

The role of perfectionism also varied across conditions. In the individual condition, high perfectionism was associated with greater anxiety and reduced wellbeing, consistent with its maladaptive effects in high-pressure solo performance contexts. In contrast, in the collaborative condition, the negative impact of perfectionism was weaker, suggesting that group contexts may help to offset the stress generated by perfectionistic standards.

Taken together, these results indicate that the psychological impact of perfectionism is not uniform but depends on the performance context. Group settings can moderate its negative consequences, whereas individual high-stakes performances may amplify them.

## General discussion

This study contributes significantly to understanding Music Performance Anxiety (MPA) and its impact on student wellbeing, particularly within the high-pressure environment of Chinese music conservatories. Consistent with previous research, both studies confirmed that MPA is negatively associated with wellbeing across multiple dimensions of the PERMA model, including Positive Emotion, Engagement, Relationships, Meaning, and Accomplishment. Specifically, high levels of anxiety were linked to reduced positive affect and diminished satisfaction in performance (Albattat, 2023; Kirsner et al., 2023). Previous studies have reported that female students and those playing certain instruments, such as bowed strings, are especially vulnerable to heightened MPA (Gül et al., 2024). Our study did not collect data on instrument type, so this factor could not be analyzed in our sample.

Study 1 extended these results by showing that perfectionism and collectivist orientation moderated the anxiety–wellbeing relationship. In this sample, higher levels of perfectionism attenuated or even reversed the negative effect of anxiety, suggesting that perfectionistic strivings may sometimes operate adaptively, providing motivation and structure (Stoeber and Otto, 2006). At the same time, prior research indicates that maladaptive forms of perfectionism can intensify anxiety and impair wellbeing when they are closely tied to self-worth (Flett and Hewitt, 2002). Collectivist orientation also played a moderating role, with students endorsing stronger collectivist values reporting a weaker negative association between anxiety and wellbeing, consistent with the protective role of social belonging and shared responsibility (Markus and Kitayama, 1991).

Study 2 experimentally examined how performance conditions shaped psychological outcomes. Students in the individual success condition reported the highest anxiety, aligning with research emphasizing that individual responsibility heightens stress in evaluative contexts (Osborne and Kirsner, 2022). Differences between individual and collaborative conditions, however, were smaller than anticipated, with collaborative settings showing only moderate benefits. For wellbeing, the pattern was more complex: unexpectedly, students in the individual condition reported higher wellbeing than those in the control group, while the collaborative condition did not differ significantly from either. This suggests that demanding individual performance contexts, although stressful, may also provide students with a sense of accomplishment or validation.

The moderating role of perfectionism varied by context. In individual settings, high perfectionism was associated with elevated anxiety and lower wellbeing, consistent with maladaptive effects documented in prior research (Racine et al., 2024; Ryan et al., 2025). By contrast, in collaborative settings, the negative impact of perfectionism was weaker, supporting findings that team or group contexts can buffer against the detrimental effects of perfectionistic strivings (Haraldsen et al., 2019; Nordin-Bates et al., 2024). Nevertheless, when group dynamics reinforce unrealistic standards, perfectionism may still contribute to performance pressure (Chen et al., 2023; Waleriańczyk, 2023). It is also important to recognize that perfectionism rarely acts in isolation. Previous studies have shown that it interacts closely with psychological factors such as self-esteem and self-efficacy, which can either buffer or amplify its effects on anxiety and wellbeing. Students with higher self-efficacy, for example, may experience perfectionistic strivings as motivating rather than distressing, while those with fragile self-esteem may be more vulnerable to maladaptive consequences. Incorporating these dimensions into future research would provide a fuller understanding of how perfectionism shapes performance-related experiences (Orejudo et al., 2017; Schillinger et al., 2021; Zhu, 2025). In addition, the cultural context of this study is highly relevant. Because collectivist values are prominent in Chinese conservatories, the results may not generalize directly to educational environments that are more individualistic. Cultural comparisons are therefore essential to determine whether the same moderation patterns between performance anxiety, perfectionism, and wellbeing emerge across diverse contexts (Hill et al., 2024; Wu and Qin, 2025).

The findings also extend prior work by showing that collectivist orientation moderated the effects of MPA. Students with strong collectivist values reported lower anxiety and higher wellbeing overall, aligning with cultural psychology frameworks that highlight the protective role of group resilience and social support (Lin, 2020). Yet collectivism was not entirely protective: in high-pressure collaborative conditions, some students experienced stress about letting the group down, reflecting research that collective obligations can also function as stressors in hierarchical collectivist contexts (Becker and Börnert-Ringleb, 2025; Cassady et al., 2024).

In conclusion, these findings emphasize that MPA does not operate in isolation but interacts with both individual difference factors (perfectionism, collectivist orientation) and contextual factors (performance conditions). Recognizing the adaptive as well as maladaptive aspects of perfectionism, and the dual role of collectivism as both buffer and potential stressor, provides a more nuanced understanding of MPA. This perspective can inform targeted interventions that support music students’ wellbeing while acknowledging the cultural and institutional contexts in which they perform (Komarenko et al., 2024).

### Limitations of the present research

Despite its contributions, this research has several limitations. First, Study 1 relied exclusively on self-reported data, which may be affected by biases such as social desirability or inaccurate self-assessment (Stoeber and Otto, 2006). Future studies could strengthen validity by incorporating complementary objective measures of stress and anxiety, such as heart rate variability or cortisol, which would provide a more comprehensive picture of the mechanisms involved (Yalcin et al., 2024).

Second, the experimental design in Study 2 was useful for isolating specific performance conditions, but it cannot fully capture the complexity of real-world performance contexts (Waleriańczyk and Stolarski, 2021). Important factors such as audience presence, adjudicator feedback, and long-term career aspirations were not directly addressed. Thus, while the scenarios were effective for testing targeted hypotheses, future studies should incorporate more ecologically valid tasks, such as live performance settings or longitudinal assessments, to enhance the applicability of the findings.

Third, the sample was drawn exclusively from music conservatories in China, where collectivist values are strongly embedded in the cultural context (Lin, 2020). While this specificity provides valuable insight into the role of collectivist orientation, it also limits the generalizability of the findings to students in more individualistic contexts. Cross-cultural studies, particularly comparing Chinese and Western conservatories, would provide an important test of whether similar moderation patterns emerge across cultures (Lin and Jackson, 2019).

In addition, the dataset did not collect information on participants’ specific specializations (e.g., vocal performance, instrumental training, composition, conducting). Since gender distributions often vary across fields, the absence of this information limits our ability to determine whether the observed gender imbalance reflects institutional patterns or was specific to this study’s sample. Future research should record these details to clarify the role of specialization in participation patterns and outcomes (Ho, 2023).

Finally, future research should expand on these limitations by incorporating more diverse student populations across cultural settings and by employing longitudinal designs (Gómez-López and Sánchez-Cabrero, 2024). Such approaches would make it possible to evaluate whether the effects observed here hold across different educational systems and to capture the long-term consequences of performance anxiety, perfectionism, and cultural orientations on students’ wellbeing and development.

#### Implications for music students and teachers

The findings of this research carry meaningful implications for both music educators and students. Since performance anxiety significantly undermines wellbeing, conservatories should consider integrating psychological training programs that equip students with effective coping strategies (Barros et al., 2024). Approaches such as mindfulness practices, cognitive restructuring, and self-compassion can help students manage anxiety more constructively and sustain their engagement with music (Kang, 2022).

Teachers also play a critical role in shaping students’ experiences of perfectionism (Sacchetti and Salustri, 2023). While maintaining high standards is central to musical excellence, placing excessive emphasis on flawlessness can have detrimental effects. Encouraging a growth mindset rather than a fixed mindset may help students interpret mistakes as part of the learning process rather than as failures, fostering resilience and persistence (Viator et al., 2024).

In addition, the moderating role of collectivist orientation underscores the value of peer support and ensemble participation. Educators can foster environments where students feel supported by one another rather than in competition, which may reduce the psychological burden of performance (Burin and Osorio, 2016). Initiatives such as group-based feedback sessions, peer mentorship, and ensemble rehearsals can reinforce a sense of shared success and belonging, helping to buffer the impact of performance-related stress.

## Conclusion

This study contributes to the existing literature by demonstrating the complex relationships among performance anxiety, wellbeing, perfectionism, and collectivist orientation in music conservatory students. Study 1 confirmed that performance anxiety was negatively associated with wellbeing, while perfectionism and collectivist orientation moderated this link in different ways. Study 2 showed that high-pressure individual performance conditions elicited the greatest anxiety, whereas collaborative and neutral settings offered partial relief. Interestingly, wellbeing was not uniformly lower in high-pressure contexts, suggesting that individual performance may also bring a sense of achievement under certain circumstances. Across both studies, perfectionism emerged as a factor with dual potential: it amplified anxiety in individual contexts but sometimes functioned adaptively when paired with social or cultural supports.

These findings highlight the necessity of balancing pressure, support, and cultural values in music education programs, so that students are challenged but not overwhelmed in their pursuit of artistic excellence. At the same time, the study’s conclusions must be interpreted with caution. The reliance on self-reported measures, the use of simulated performance conditions, and the cultural specificity of the Chinese conservatory context all limit the direct generalizability of the findings. Future research should therefore incorporate more diverse student populations, include objective physiological indicators of anxiety, and adopt longitudinal designs to capture the longer-term effects of performance-related stress.

By acknowledging both the contributions and the limitations of the present work, the study offers a nuanced perspective on how psychological and cultural factors intersect in music education. Promoting adaptive forms of perfectionism, strengthening social support, and fostering resilience can help institutions create learning environments where students thrive both artistically and emotionally.

## Data Availability

The datasets presented in this study can be found in online repositories. The dataset used for this research is stored at OSF: https://tinyurl.com/MPA-2-studies.
